# Perioperative Thyroid-Metabolic Changes in Pancreatic Ductal Adenocarcinoma According to Surgical Management

**DOI:** 10.3390/cancers18111769

**Published:** 2026-05-28

**Authors:** Oliwia Grząsiak-Kraj, Tomasz Kraj, Alicja Majos, Aleksander Wardęszkiewicz, Aneta Szmiel, Krzysztof Poznański, Adam Durczyński, Piotr Hogendorf, Janusz Strzelczyk

**Affiliations:** 1 Department of General and Transplant Surgery, Medical University of Lodz, 90-419 Lodz, Poland; 2 Department of Vascular Surgery and Angiology, Independent Public Healthcare Institution of the Ministry of the Interior and Administration in Lodz, 91-425 Lodz, Poland; 3 Institute of Medical Expertise in Lodz, 91-205 Lodz, Poland

**Keywords:** pancreatic ductal adenocarcinoma, thyroid hormones, FT3/FT4 ratio, perioperative changes, metabolic response, nutritional status, pancreatic surgery, low-T3 syndrome

## Abstract

**Background:** Pancreatic ductal adenocarcinoma (PDAC) is associated not only with tumor progression but also with profound metabolic and nutritional disturbances. Thyroid hormone homeostasis may reflect this systemic response; however, perioperative data in PDAC remain limited. We aimed to assess perioperative changes in thyroid-related parameters in patients with PDAC undergoing different types of surgical management and to explore their associations with nutritional, metabolic, and tumor-burden variables. **Methods:** We performed a retrospective single-center study including 101 patients with PDAC. Thyroid-related and metabolic laboratory parameters were assessed before surgery and again 4–6 weeks later. The analyzed variables included thyroid-stimulating hormone (TSH), free triiodothyronine (FT3), free thyroxine (FT4), the FT3/FT4 ratio, albumin, total protein, glucose, insulin, HbA1c, lipid parameters, and CA 19-9. Patients were analyzed according to resectional versus non-resectional treatment and according to four procedure types. The primary endpoint was perioperative change in the FT3/FT4 ratio. **Results:** At baseline, resectional patients had significantly higher FT3 and FT3/FT4 ratio values and lower FT4 and CA 19-9 levels than non-resectional patients. In the whole cohort, FT3 and the FT3/FT4 ratio decreased significantly after treatment, whereas TSH increased, and FT4 remained unchanged. These endocrine changes occurred in parallel with significant declines in albumin, total protein, glucose, insulin, HbA1c, and HDL cholesterol, together with an increase in triglyceride levels. Baseline FT3 and FT3/FT4 ratios correlated positively with albumin and total protein and negatively with CA 19-9. Although perioperative changes did not differ significantly between resectional and non-resectional groups except for triglycerides, significant procedure-dependent differences were observed across the four surgical categories for FT3, FT4, TSH, and the FT3/FT4 ratio; glucose; insulin; and triglycerides. The prevalence of low-T3 syndrome increased from 11.1% preoperatively to 38.7% postoperatively. **Conclusions:** In PDAC, perioperative changes in thyroid hormone indices are pronounced and strongly depend on the type of surgical management. FT3 and the FT3/FT4 ratio appear to reflect systemic metabolic and nutritional adaptation as well as disease burden rather than acting as tumor-specific markers.

## Simple Summary

Pancreatic ductal adenocarcinoma (PDAC) is associated not only with tumor progression but also with profound metabolic and nutritional disturbances. Thyroid hormone metabolism may reflect this systemic response, yet perioperative changes in thyroid-related parameters in PDAC remain insufficiently characterized. In this study, we assessed free triiodothyronine (FT3), free thyroxine (FT4), thyroid-stimulating hormone (TSH), and the FT3/FT4 ratio before surgery and again 4–6 weeks later in patients undergoing different types of surgical management. We found that FT3 and the FT3/FT4 ratio decreased significantly after treatment, while the magnitude and pattern of change differed according to procedure type. Baseline thyroid-related indices were also associated with nutritional status and tumor burden. These findings suggest that thyroid-related parameters in PDAC may be better interpreted as markers of systemic metabolic adaptation than as tumor-specific markers.

## 1. Introduction

Pancreatic ductal adenocarcinoma (PDAC) remains one of the most lethal solid malignancies and continues to present major challenges in diagnosis, resectability assessment, and treatment selection [1,2].

Beyond local tumor progression, PDAC is frequently accompanied by cachexia, progressive nutritional deterioration, and metabolic derangements that substantially affect patient condition and outcomes [2,3,4].

Accordingly, biomarkers reflecting systemic host response rather than tumor burden alone may provide clinically relevant information in this disease [2,3,4].

Among the endocrine pathways potentially involved in this systemic response, thyroid hormone homeostasis is of particular interest because peripheral conversion of thyroxine (T4) to biologically active triiodothyronine (T3) is regulated by tissue deiodinases and closely linked to cellular energy metabolism [5,6].

Disturbances in this axis may arise not only in primary thyroid disease but also in cancer, inflammation, starvation, and critical illness [5,6,7].

The resulting pattern, commonly described as non-thyroidal illness syndrome (NTIS) or low-T3 syndrome, is typically characterized by reduced T3 concentrations with normal or low–normal T4 concentrations and an absent or blunted compensatory TSH response, and it has been associated with adverse outcomes in severe systemic illness [7,8,9].

The relationship between thyroid hormones and cancer is biologically plausible but clinically complex [6,10].

Experimental and translational studies suggest that thyroid hormone signaling may influence proliferation, invasion, angiogenesis, and tumor microenvironment interactions, partly through non-genomic pathways involving integrin αvβ3 [10,11,12]. At the same time, circulating thyroid-related indices such as FT3, FT4, and the FT3/FT4 ratio may also reflect nutritional reserve, inflammatory burden, and broader metabolic adaptation rather than direct tumor-specific activity alone [6,8,10,13].

In pancreatic cancer, however, the available evidence remains limited [13,14,15]. A preliminary study from our center demonstrated differences between portal and peripheral thyroid hormone concentrations in patients with pancreatic cancer, suggesting a link between FT3-related parameters and the local neoplastic process [13]. In a subsequent pilot study of radically resected PDAC, a lower preoperative FT3/FT4 ratio was associated with shorter overall survival [14].

Additional clinical data indicate that thyroid disorders and thyroid hormone exposure may coexist with PDAC, but their clinical relevance remains uncertain [15].

This question is especially relevant in surgical oncology because pancreaticoduodenectomy, distal pancreatectomy, palliative bypass, and exploratory laparotomy represent biologically and metabolically distinct clinical scenarios [1,4,16,17]. Previous studies have shown that glucose metabolism and endocrine adaptation after pancreatectomy differ substantially according to the type of resection, supporting the concept that procedure-specific metabolic trajectories should be analyzed separately [16,17].

However, longitudinal perioperative data specifically addressing thyroid-related changes across different surgical pathways in PDAC remain scarce [13,14,15,16,17].

Therefore, the aim of the present study was to evaluate perioperative changes in thyroid hormone homeostasis in patients with PDAC undergoing different types of surgical management [13,14,15,16,17,18]. Specifically, we assessed serum thyroid-stimulating hormone (TSH), free triiodothyronine (FT3), free thyroxine (FT4), and the FT3/FT4 ratio before treatment and at early postoperative follow-up, and we analyzed their relationships with metabolic, nutritional, and selected tumor-related laboratory parameters [13,14,15,16,17,18]. We hypothesized that perioperative thyroid-related indices, particularly FT3 and the FT3/FT4 ratio, reflect clinically meaningful thyroid-metabolic adaptation in PDAC and may serve as biomarkers of systemic host response rather than tumor-specific markers [6,8,10,13,14,15,16,17,18].

## 2. Materials and Methods

### 2.1. Study Design and Patients

This retrospective single-center cohort study included 101 adult patients with pancreatic ductal adenocarcinoma (PDAC) who underwent surgical exploration at the Department of General and Transplant Surgery, Medical University of Lodz, Lodz, Poland. Patients were identified from consecutive cases treated between January 2021 and December 2025. The final analytic cohort consisted exclusively of patients included in the cleaned dataset used for the definitive statistical analysis.

Of the 101 patients, 49 underwent resectional treatment, and 52 underwent non-resectional treatment. Within the resectional group, 22 patients underwent pancreaticoduodenectomy (Whipple procedure), and 27 underwent distal pancreatectomy with splenectomy. Within the non-resectional group, 25 patients underwent palliative bypass procedures, and 27 underwent exploratory laparotomy. In the non-resectional group, the diagnosis of PDAC was confirmed histopathologically on the basis of core-needle biopsy or surgical biopsy specimens.

The inclusion criteria were as follows: adult patients with histologically confirmed pancreatic ductal adenocarcinoma, surgical exploration or surgical treatment performed at the Department of General and Transplant Surgery, Medical University of Lodz, availability of baseline thyroid-related laboratory parameters, and availability of clinical data required for group allocation and statistical analysis. In addition, the final analytic cohort was restricted to patients with postoperative complications not exceeding Clavien–Dindo grade IIIa.

The exclusion criteria were: lack of histopathological confirmation of PDAC; incomplete data preventing classification into surgical groups; absence of baseline thyroid-related laboratory measurements; active or uncontrolled thyroid dysfunction at the time of surgery; and initiation or recent modification of thyroid-related hormonal therapy, including levothyroxine, during the perioperative observation period. Patients with severe postoperative complications requiring reintervention under general anesthesia, intensive care management for organ failure, or resulting in death (Clavien–Dindo grades IIIb–V) were excluded from the final analytic cohort.

Patients with a documented history of thyroid disease were not excluded if the condition was clinically stable and there was no evidence of uncontrolled thyroid dysfunction or recent modification of thyroid-related therapy during the perioperative observation period. Thyroid disease was therefore retained as a clinical covariate and was included in the propensity score-adjusted sensitivity analysis.

Medication records were reviewed for thyroid-related therapy and selected thyroid-active medications. Patients treated for hypothyroidism received levothyroxine and were retained in the analysis only if the condition was clinically stable and no initiation or dose modification of thyroid-related hormonal therapy occurred during the perioperative observation period. No documented use of amiodarone or systemic glucocorticoids was identified in the medical records of the analyzed patients. Exposure to iodine-containing contrast could not be reliably excluded because contrast-enhanced imaging is routinely used in the diagnostic and staging work-up of PDAC.

**Figure 1:**
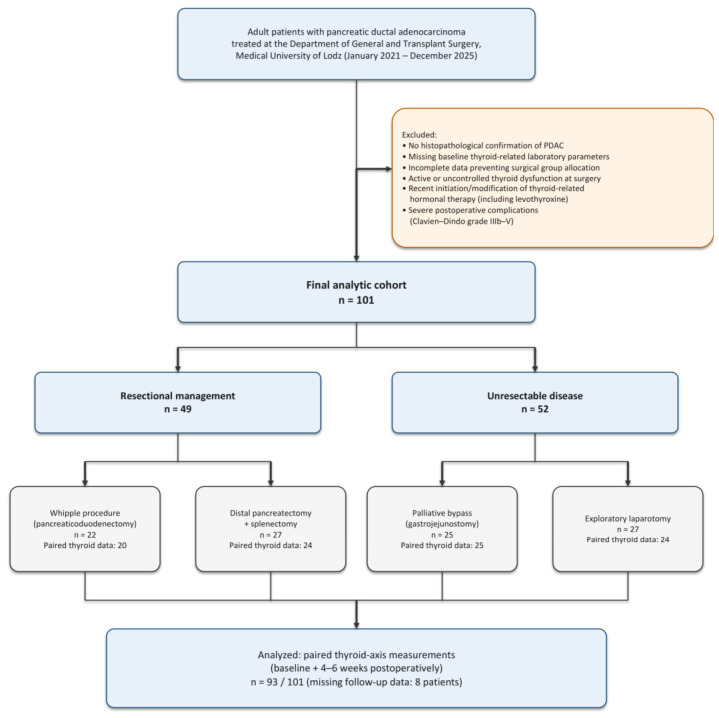
Participant flow diagram. PDAC: pancreatic ductal adenocarcinoma.

Laboratory parameters were assessed at two predefined time points: preoperatively (baseline) and at routine follow-up 4–6 weeks after surgery. Postoperative laboratory measurements were obtained during routine clinical follow-up 4–6 weeks after surgery and not during acute readmission or ongoing inpatient management of postoperative complications. As described above, patients with severe postoperative complications corresponding to Clavien–Dindo grades IIIb–V were not included in the final analytic cohort. Thyroid-related parameters were available at baseline in 99/101 patients and at follow-up in 93/101 patients. Because the availability of paired laboratory measurements differed across variables, the effective sample size varied between analyses. The participant flow diagram is shown in Figure 1.

### 2.2. Study Groups and Definitions

Patients were analyzed using two complementary grouping strategies. First, they were categorized as resectional or non-resectional in order to compare patients who underwent tumor resection with those managed without resection. Second, for procedure-specific analyses, patients were classified into four surgical groups: Whipple procedure, distal pancreatectomy with splenectomy, palliative bypass, and exploratory laparotomy.

The FT3/FT4 ratio was calculated at both time points as the quotient of serum-free triiodothyronine (FT3) and free thyroxine (FT4) and was used as an indirect index of peripheral thyroid hormone conversion. Perioperative change was defined as the difference between postoperative and preoperative values and was expressed as Δ = postoperative value − preoperative value.

Low-T3 syndrome was defined as FT3 < 2.0 pg/mL with TSH within the range of 0.3–4.0 mIU/L. This more stringent FT3 threshold was selected to identify a clinically pronounced low-T3 phenotype rather than borderline values below the local laboratory reference range. The TSH criterion was used to exclude overt primary thyroid dysfunction and to capture a low-T3 pattern occurring without an appropriate compensatory TSH increase. Because reverse T3 was not measured, this phenotype was not considered a definitive biochemical diagnosis of non-thyroidal illness syndrome (NTIS), but rather a low-T3 phenotype compatible with NTIS in the appropriate clinical context [7,8,9].

### 2.3. Data Collection and Laboratory Variables

Clinical and laboratory data were collected retrospectively from medical records. The analyzed variables included age, selected comorbidities, type of surgical management, and laboratory parameters measured at baseline and follow-up.

The laboratory variables included: TSH, FT3, FT4, albumin, total protein, glucose, insulin, glycated hemoglobin (HbA1c), total cholesterol, low-density lipoprotein cholesterol (LDL), high-density lipoprotein cholesterol (HDL), triglycerides (TG), and carbohydrate antigen 19-9 (CA 19-9). The reference ranges for thyroid-related parameters were as follows: TSH, 0.38–5.33 µU/mL; FT3, 2.5–4.3 pg/mL; and FT4, 0.61–1.12 ng/dL.

Serum FT3 and FT4 concentrations were measured using a UniCel DxI 800 analyzer (Beckman Coulter, Inc., Brea, CA, USA). During the assays, FT3 and FT4 were also assessed in internal quality-control material, allowing evaluation of analytical precision. The coefficient of variation in the control material was 4.72% for FT3 (SD 0.202 pg/mL) and 3.70% for FT4 (SD 0.159 ng/dL). All measurements were performed according to routine hospital laboratory procedures and internal quality-control standards.

The FT3/FT4 ratio was calculated for each patient at both time points. In addition, perioperative Δ values were calculated for thyroid-related, nutritional, metabolic, and tumor-marker parameters.

### 2.4. Study Endpoints

The primary endpoint of the study was perioperative change in the FT3/FT4 ratio between baseline and postoperative follow-up.

The secondary endpoints were: perioperative changes in TSH, FT3, and FT4; perioperative changes in albumin, total protein, glucose, insulin, HbA1c, and lipid parameters; baseline differences in thyroid-related, nutritional, metabolic, and tumor-related parameters between resectional and non-resectional patients; baseline correlations between FT3 or FT3/FT4 ratio and selected clinical or laboratory variables; comparison of perioperative changes between resectional vs. non-resectional groups and across the four surgical groups; perioperative prevalence and redistribution of low-T3 syndrome; and exploratory evaluation of the associations of FT3 and the FT3/FT4 ratio with resectional versus non-resectional status using logistic regression and ROC-based analyses.

### 2.5. Statistical Analysis

Because the distributions of the analyzed variables deviated from normality on Shapiro–Wilk testing and subgroup sizes were small, non-parametric methods were used. Continuous variables are presented as medians and interquartile ranges (IQRs), whereas categorical variables are presented as counts and percentages.

Differences between two independent groups (resection vs. non-resection) were assessed using the Mann–Whitney U test for continuous variables and Fisher’s exact test for categorical variables. Perioperative within-patient changes (baseline vs. follow-up at 4–6 weeks) were assessed using the Wilcoxon signed-rank test for paired observations. Comparisons across the four surgical groups were performed using the Kruskal–Wallis test. Associations between continuous variables were evaluated using Spearman’s rank correlation coefficient (ρ).

Because CA 19-9 showed a markedly right-skewed distribution, it was analyzed after log(1 + x) transformation in correlation and regression analyses. Differences in the prevalence of low-T3 syndrome between baseline and follow-up were assessed using McNemar’s test.

Exploratory associations with resectional versus non-resectional status were evaluated using univariable and multivariable logistic regression models, with model fit assessed using the Akaike information criterion (AIC) and pseudo-R^2^. The exploratory discriminative performance of the FT3/FT4 ratio and isolated FT3 measurement for distinguishing resectional from non-resectional status was evaluated using receiver operating characteristic (ROC) analysis, with comparison of area under the curve (AUC) values performed using a bootstrap test with 5000 repetitions.

A propensity score-adjusted sensitivity analysis was performed to assess the robustness of the main findings. The propensity score was estimated by logistic regression of resectional status on age, diabetes, arterial hypertension, thyroid disease, and dyslipidemia; postoperative or mediator-related variables (thyroid-axis parameters, nutritional and metabolic markers, CA 19-9) were excluded by design. Inverse-probability-of-treatment weighting (IPTW) was applied, with weights truncated at the 1st and 99th percentile. Covariate balance before and after weighting was assessed using standardized mean differences (Supplementary Table S1A); IPTW-weighted between-group contrasts for the principal outcomes are reported in Supplementary Table S1B.

The significance level was set at α = 0.05. All analyses were performed in R v4.3 using the tidyverse and ggpubr packages.

The study was designed around a single pre-specified primary endpoint—the perioperative change in the FT3/FT4 ratio. All other analyses were designated a priori as secondary or exploratory and are presented as hypothesis-generating. To assess the impact of multiplicity on the principal family of paired pre-vs-post tests in the whole cohort, *p* values were additionally adjusted using the Benjamini–Hochberg false discovery rate (BH-FDR) procedure. The same procedure was applied to the families of four-group Kruskal–Wallis tests and to the principal Spearman correlation analyses, with results reported in Supplementary Table S2. For post hoc pairwise comparisons after significant Kruskal–Wallis tests across the four surgical groups, Dunn’s test with Bonferroni correction across the six pairwise contrasts per family was used (Supplementary Table S3).

### 2.6. Missing Data Handling

All analyses were performed using available-case data for each variable. Accordingly, the number of observations differed across individual tests depending on the availability of baseline values, follow-up values, and paired measurements. The effective sample size for each analysis is reported in the corresponding Section 3 tables.

Missing postoperative laboratory values may not have been completely random. In this retrospective surgical cohort, absence of follow-up measurements may have reflected postoperative morbidity, delayed outpatient follow-up, readmission to another hospital, loss to follow-up, or other clinical factors affecting the availability of laboratory reassessment. Therefore, although available-case analysis was used, the possibility of informative missingness cannot be excluded and should be considered when interpreting perioperative changes.

The number of available and missing observations for each variable and time point is reported in Supplementary Table S4.

## 3. Results

### 3.1. Baseline Characteristics

The final analytic cohort included 101 patients with pancreatic ductal adenocarcinoma (PDAC), of whom 49 underwent resectional treatment and 52 underwent non-resectional treatment. The resectional group comprised 22 patients treated with the Whipple procedure and 27 treated with distal pancreatectomy with splenectomy, whereas the non-resectional group comprised 25 patients treated with palliative bypass and 27 who underwent exploratory laparotomy. Thyroid-related parameters were available at baseline in 99/101 patients and at follow-up in 93/101 patients.

Already at baseline, the two groups differed not only in tumor burden but also in hormonal and metabolic profile. Patients in the resectional group had significantly higher FT3 concentrations than those in the non-resectional group (median 2.8 vs. 2.4 pg/mL, *p* = 0.003) and significantly lower FT4 concentrations (1.1 vs. 1.4 ng/dL, *p* = 0.002). Consequently, the FT3/FT4 ratio was markedly higher in resectional patients (2.4 vs. 1.7, *p* < 0.001), suggesting more preserved peripheral thyroid hormone conversion in patients qualified for radical surgery.

**Figure 2:**
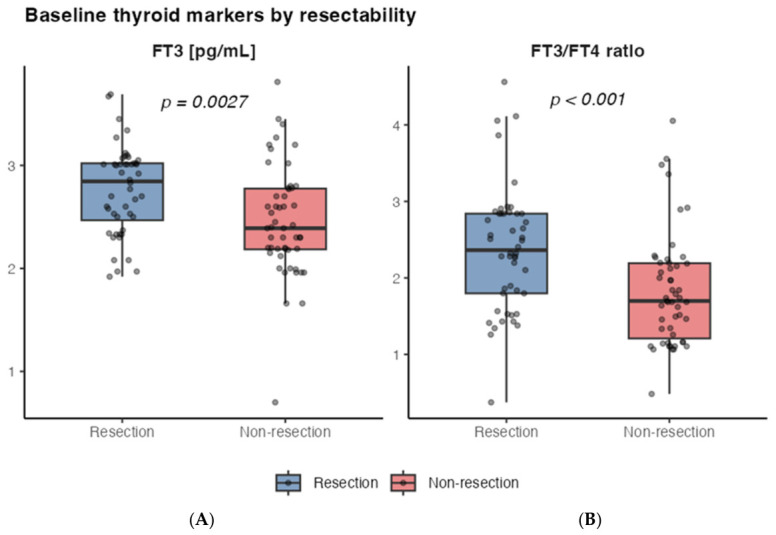
Baseline FT3 and FT3/FT4 ratio according to resectability. Panel (**A**) shows FT3, and panel (**B**) shows the FT3/FT4 ratio in resectional and non-resectional patients. Boxplots show the median, interquartile range, and whiskers; overlaid dots represent individual patients. The displayed *p* values are from the Mann–Whitney U test.

This pattern was accompanied by a more favorable nutritional and metabolic profile. The resectional group had higher albumin (42.7 vs. 38.0 g/L, *p* = 0.001) and total protein concentrations (71.0 vs. 62.4 g/L, *p* = 0.031), higher HDL cholesterol (45.0 vs. 37.5 mg/dL, *p* = 0.028), lower triglycerides (84.0 vs. 114.5 mg/dL, *p* = 0.011), and lower HbA1c (5.5% vs. 5.8%, *p* = 0.024). CA 19-9 concentrations differed by approximately one order of magnitude between groups (7.9 vs. 77.7 U/mL, *p* < 0.001). In contrast, age, glucose, insulin, LDL cholesterol, total cholesterol, and the prevalence of diabetes, thyroid disease, arterial hypertension, and dyslipidemia did not differ significantly between groups (Table 1 and Figure 2).

### 3.2. Baseline Correlations of Thyroid-Related Parameters with Systemic Status

To determine whether the association between thyroid-related parameters and resectability reflected broader systemic condition, baseline correlations were examined.

Baseline FT3 showed significant positive correlations with markers of nutritional status, including albumin (ρ = +0.356, *p* < 0.001) and total protein (ρ = +0.283, *p* = 0.005). A similar, although weaker, pattern was observed for the FT3/FT4 ratio, which correlated positively with albumin (ρ = +0.212, *p* = 0.037) and total protein (ρ = +0.206, *p* = 0.044). Both thyroid-related parameters correlated negatively with log-transformed CA 19-9 (FT3: ρ = −0.320, *p* = 0.001; FT3/FT4: ρ = −0.329, *p* < 0.001) and with age (FT3: ρ = −0.285, *p* = 0.004; FT3/FT4: ρ = −0.238, *p* = 0.018).

**Figure 3:**
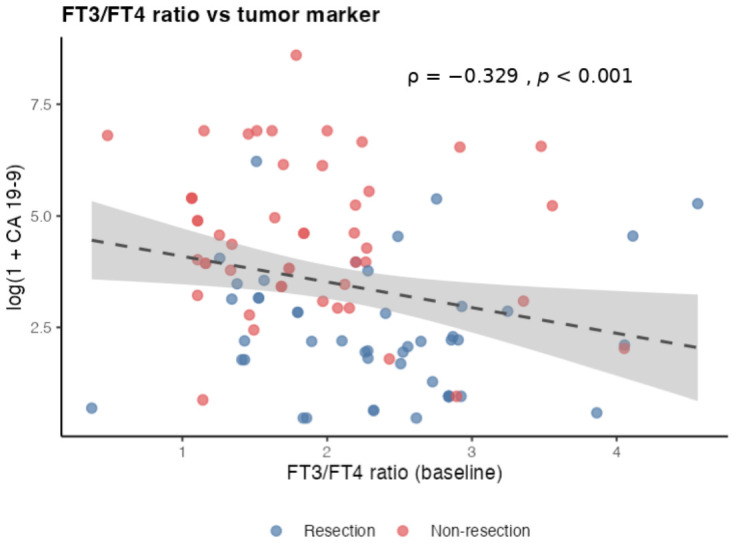
Association between baseline FT3/FT4 ratio and log-transformed CA 19-9. Each dot represents one patient; blue dots indicate resectional patients, and red dots indicate non-resectional patients. The dashed line represents the fitted linear trend, and the gray shaded area indicates its 95% confidence interval. The figure displays the Spearman correlation coefficient (ρ) and corresponding *p* value.

**Figure 4:**
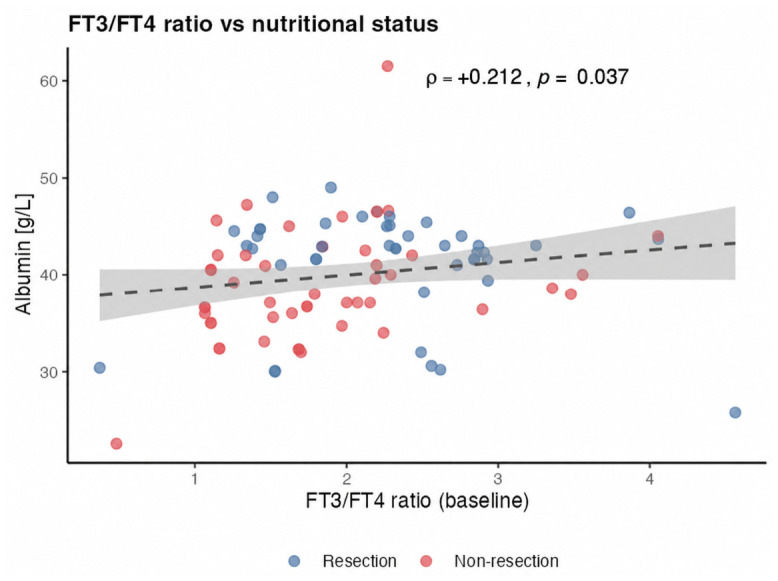
Association between baseline FT3/FT4 ratio and albumin. Each dot represents one patient; blue dots indicate resectional patients, and red dots indicate non-resectional patients. The dashed line represents the fitted linear trend, and the gray shaded area indicates its 95% confidence interval. The figure displays the Spearman correlation coefficient (ρ) and corresponding *p* value.

Importantly, baseline FT3 did not correlate significantly with glucose (ρ = −0.156, *p* = 0.123), insulin (ρ = −0.043, *p* = 0.674), HbA1c (ρ = +0.011, *p* = 0.914), HDL cholesterol (*p* = 0.074), or triglycerides (*p* = 0.243). These findings indicate that, before surgery, thyroid-related indices were associated primarily with nutritional status and tumor burden rather than with baseline carbohydrate or lipid metabolism (Table 2; Figure 3, Figure 4 and Figure S1).

### 3.3. Perioperative Changes in the Whole Cohort

In the whole cohort, surgery was followed by significant changes in several thyroid-related, nutritional, and metabolic parameters. FT3 concentrations decreased significantly from baseline to follow-up (Δ median = −0.65 pg/mL, *p* < 0.001), and the FT3/FT4 ratio also declined significantly (Δ = −0.43, *p* < 0.001). In contrast, FT4 concentrations did not change significantly (*p* = 0.110), whereas TSH increased significantly (Δ = +0.28, *p* < 0.001).

These endocrine changes occurred in parallel with marked deterioration in nutritional parameters. Albumin decreased by a median of 8.65 g/L (*p* < 0.001) and total protein by 7.10 g/L (*p* < 0.001). Significant metabolic changes were also observed: glucose decreased by 14.5 mg/dL (*p* < 0.001), insulin by 1.26 μIU/mL (*p* < 0.001), HbA1c by 0.20% (*p* < 0.001), and HDL cholesterol by 8.0 mg/dL (*p* < 0.001), whereas triglycerides increased by 36.0 mg/dL (*p* < 0.001). Total cholesterol also decreased significantly (Δ = −12.0 mg/dL, *p* = 0.011), while LDL cholesterol did not change significantly (*p* = 0.080) (Table 3).

A key finding of the perioperative correlation analysis was that the decline in the FT3/FT4 ratio correlated significantly with decreases in glucose (ρ = −0.225, *p* = 0.030) and insulin (ρ = −0.219, *p* = 0.040). In contrast, isolated changes in FT3 were not significantly correlated with metabolic variables. These findings suggest that changes in the FT3/FT4 ratio may provide complementary information on perioperative thyroid-metabolic adaptation; however, this should be interpreted cautiously given the exploratory nature of these analyses (Table 3 and Figure S2).

### 3.4. Procedure-Dependent Thyroid-Metabolic Response

**Figure 5:**
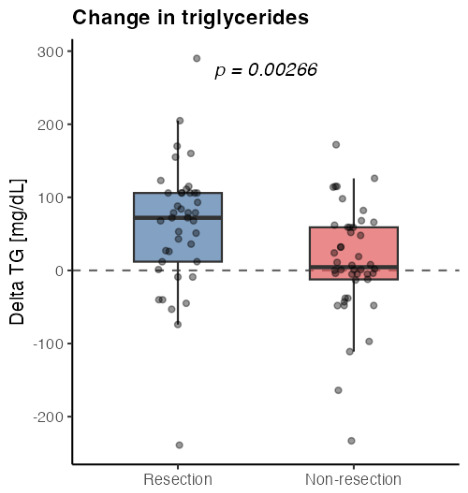
Perioperative change in triglycerides according to resectional versus non-resectional treatment. Boxplots show the median, interquartile range, and whiskers; overlaid dots represent individual patients. The horizontal dashed line marks no change (Δ = 0), where Δ denotes postoperative minus preoperative value. The *p* value refers to the between-group comparison by Mann–Whitney U test.

When patients were grouped broadly as resectional versus non-resectional, no significant between-group differences were observed in the magnitude of perioperative thyroid-related changes, including ΔFT3 (*p* = 0.110), ΔFT3/FT4 (*p* = 0.188), and ΔTSH (*p* = 0.114). Similarly, most metabolic changes did not differ significantly between the two groups, including Δalbumin (*p* = 0.749) and Δglucose (*p* = 0.748). The only significant exception was triglycerides, which increased more strongly in the resectional group than in the non-resectional group (ΔTG: +72.0 vs. +4.5 mg/dL, *p* = 0.003) (Table 4A and Figure 5).

Perioperative changes are presented separately for the broad two-group comparison between resectional and non-resectional treatment (Table 4A) and for the four-group procedure-specific comparison (Table 4B).

**Figure 6:**
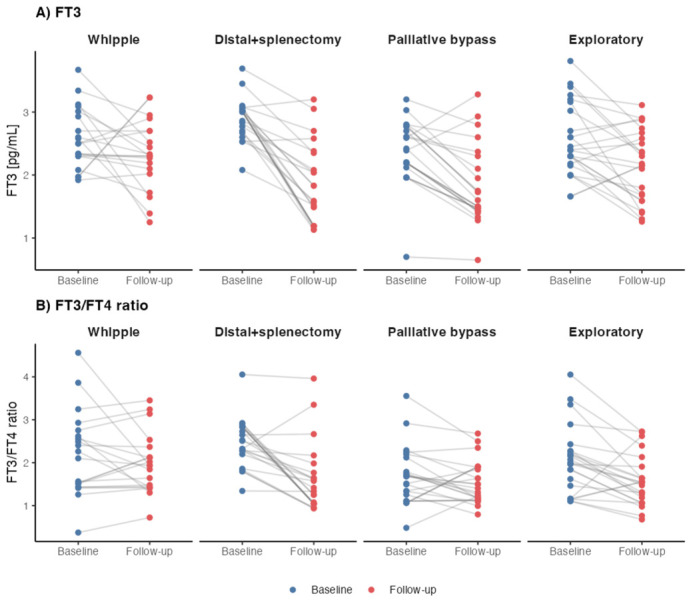
Paired perioperative trajectories of FT3 and the FT3/FT4 ratio across four surgical groups. Panel (**A**) shows FT3 and Panel (**B**) shows the FT3/FT4 ratio. Each dot represents one patient, and gray lines connect paired baseline and follow-up values within the same individual. Blue dots indicate baseline measurements, and red dots indicate follow-up measurements. Groups are shown from left to right as Whipple procedure, distal pancreatectomy with splenectomy, palliative bypass, and exploratory laparotomy.

**Figure 7:**
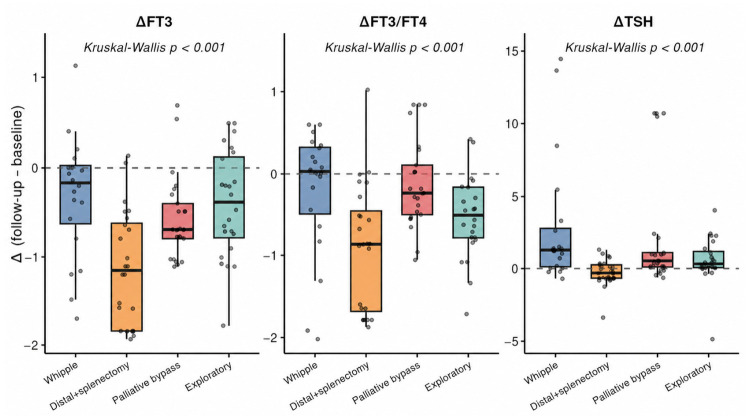
Distribution of perioperative changes in FT3, FT3/FT4 ratio, and TSH according to surgical group. Boxplots show the median, interquartile range, and whiskers; overlaid dots represent individual patients. The horizontal dashed line marks no change (Δ = 0), where Δ denotes postoperative minus preoperative value. Reported *p* values are from the Kruskal–Wallis test comparing the four surgical groups.

**Figure 8:**
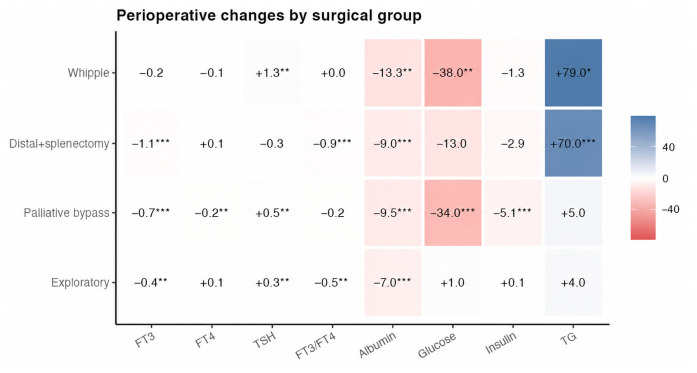
Heatmap of perioperative changes in thyroid-related, nutritional, and metabolic parameters by surgical group. Each cell shows the median perioperative change (Δ = postoperative minus preoperative value) for the indicated parameter and procedure type. Blue shading indicates positive changes, and red shading indicates negative changes, with deeper color intensity corresponding to larger absolute changes. Asterisks indicate within-group significance levels (* *p* < 0.05, ** *p* < 0.01, *** *p* < 0.001).

However, this broad two-group comparison masked substantial heterogeneity between procedures. Kruskal–Wallis analysis across the four surgical groups revealed significant differences for ΔFT3 (*p* < 0.001), ΔFT4 (*p* = 0.003), ΔTSH (*p* < 0.001), ΔFT3/FT4 (*p* < 0.001), Δglucose (*p* < 0.001), Δinsulin (*p* < 0.001), and ΔTG (*p* = 0.027) (Table 4B and Figure 6, Figure 7 and Figure 8).

**Figure 9:**
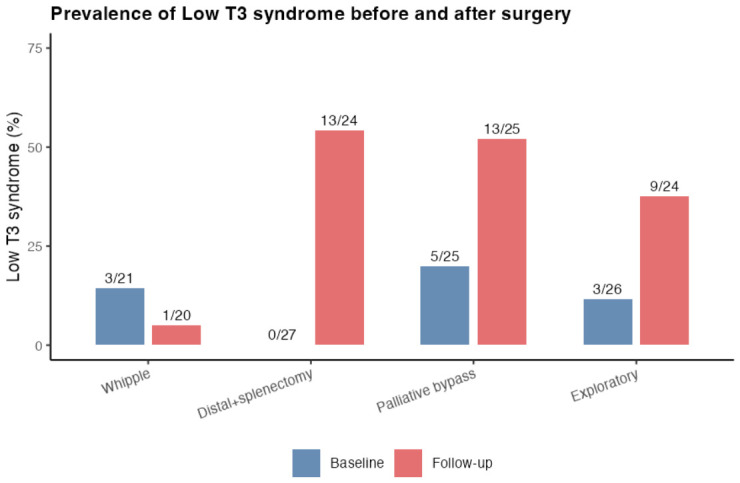
Prevalence of low-T3 syndrome before and after surgery according to surgical group. Blue bars indicate baseline values, and red bars indicate follow-up values. Numbers above the bars show the number of patients with low-T3 syndrome over the total number assessed in each subgroup. Low-T3 syndrome was defined as FT3 < 2.0 pg/mL with TSH 0.3–4.0 mIU/L.

Patients undergoing the Whipple procedure showed a distinctive profile. Despite the largest decrease in albumin (Δ = −13.35 g/L) and a marked decline in glucose (Δ = −38.0 mg/dL), FT3 concentrations remained relatively stable (Δ = −0.17, *p* = 0.127), and the FT3/FT4 ratio did not change significantly (Δ = +0.03, *p* = 0.808). At the same time, this group showed the strongest increase in TSH (Δ = +1.27, *p* = 0.001). Consistent with this pattern, postoperative low-T3 syndrome was rare after the Whipple procedure (5.0%) (Table 4B and Figure 6, Figure 7, Figure 8 and Figure 9).

In contrast, distal pancreatectomy with splenectomy was associated with the most pronounced decline in FT3 (Δ = −1.15, *p* < 0.001) and FT3/FT4 ratio (Δ = −0.86, *p* < 0.001), without a compensatory rise in TSH (Δ = −0.31, *p* = 0.178). This subgroup also had the highest prevalence of postoperative low-T3 syndrome (54.2%) (Table 4B and Figure 6, Figure 7, Figure 8 and Figure 9).

Palliative bypass procedures showed an intermediate but mixed profile, with decreases in FT3 (Δ = −0.69, *p* < 0.001) and FT4 (Δ = −0.23, *p* = 0.003), together with an increase in TSH (Δ = +0.52, *p* = 0.001). This group also showed the largest decline in insulin (Δ = −5.10, *p* < 0.001), and postoperative low-T3 syndrome occurred in 52.0% of patients (Table 4B and Figure 6, Figure 7, Figure 8 and Figure 9).

Exploratory laparotomy was associated with a moderate decrease in FT3 (Δ = −0.39, *p* = 0.006) and FT3/FT4 ratio (Δ = −0.51, *p* = 0.001), together with an increase in TSH (Δ = +0.32, *p* = 0.004). In this subgroup, glucose (Δ = +1.0, *p* = 0.981) and insulin (Δ = +0.08, *p* = 0.666) remained largely unchanged. Postoperative low-T3 syndrome occurred in 37.5% of patients (Table 4B and Figure 6, Figure 7, Figure 8 and Figure 9).

### 3.5. Low-T3 Syndrome

The prevalence of low-T3 syndrome increased significantly after surgery, from 11.1% (11/99) at baseline to 38.7% (36/93) at follow-up (McNemar’s test: χ^2^ = 12.80, *p* < 0.001). Thus, the frequency of this pattern increased nearly fourfold in the perioperative setting (Table 5A and Figure 9).

Transition analysis demonstrated a bidirectional change in thyroid status. Among 82 patients with normal baseline FT3, 35 (43%) developed low-T3 syndrome de novo after surgery. Conversely, 10 of 11 patients (91%) with preoperative low-T3 normalized at follow-up (Table 5B).

This bidirectional transition indicates that preoperative and postoperative low-T3 status did not simply represent persistence of the same phenotype. Rather, most postoperative low-T3 cases developed de novo among patients with normal baseline FT3, whereas most patients with preoperative low-T3 no longer fulfilled the low-T3 definition at follow-up.

The distribution of postoperative low-T3 syndrome differed markedly between surgical groups. The lowest prevalence was observed after the Whipple procedure (5.0%), whereas the highest prevalence occurred after distal pancreatectomy with splenectomy (54.2%) and palliative bypass (52.0%). Exploratory laparotomy showed an intermediate prevalence of 37.5% (Table 5C and Figure 9).

**Figure 10:**
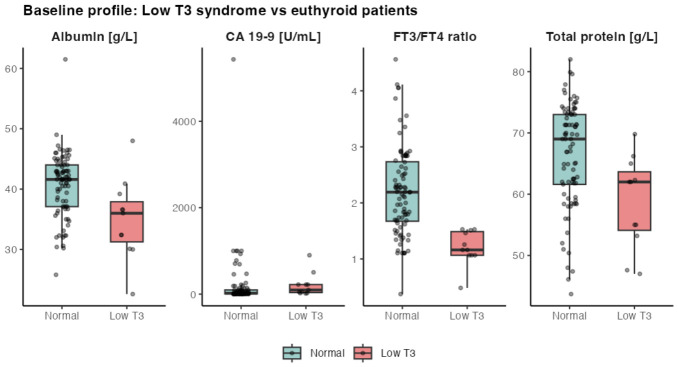
Baseline profile of patients with low-T3 syndrome versus euthyroid patients. The four panels show albumin, CA 19-9, FT3/FT4 ratio, and total protein. Boxplots show the median, interquartile range, and whiskers; overlaid dots represent individual patients. Low-T3 syndrome was defined as FT3 < 2.0 pg/mL with TSH 0.3–4.0 mIU/L.

Patients with preoperative low-T3 syndrome also had a significantly less favorable baseline profile than euthyroid patients, including lower albumin (36.0 vs. 41.6 g/L, *p* = 0.003), lower total protein (62.0 vs. 69.0 g/L, *p* = 0.006), higher CA 19-9 (95.7 vs. 21.0 U/mL, *p* = 0.020), and lower FT3/FT4 ratio results (1.2 vs. 2.2, *p* < 0.001) (Table 5D and Figure 10).

### 3.6. Exploratory Association of the FT3/FT4 Ratio with Resectional Status

**Figure 11:**
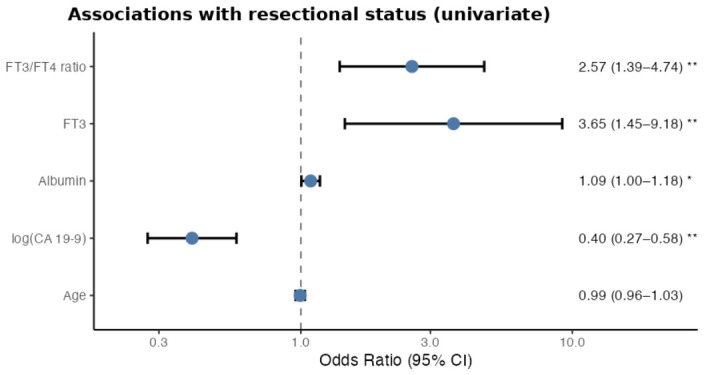
Univariable associations with resectional status. The forest plot shows odds ratios (ORs) with 95% confidence intervals from univariable logistic regression models on a logarithmic scale. The vertical dashed line indicates OR = 1. Variables to the right of this line are associated with higher odds of belonging to the resectional group, whereas variables to the left are associated with lower odds of belonging to the resectional group. Asterisks indicate statistical significance in univariate logistic regression: * *p* < 0.05; ** *p* < 0.01.

In univariable logistic regression, both the FT3/FT4 ratio (OR = 2.57, 95% CI: 1.39–4.74, *p* = 0.003) and FT3 concentration (OR = 3.65, 95% CI: 1.45–9.18, *p* = 0.006) were positively associated with resectional status. Albumin was also associated with resectional status (OR = 1.09, *p* = 0.038), whereas log(CA 19-9) showed the strongest inverse association (OR = 0.40, 95% CI: 0.27–0.58, *p* < 0.001). Age was not associated with resectional status (OR = 0.99, *p* = 0.786) (Table 6A and Figure 11).

In multivariable models including CA 19-9, the FT3/FT4 ratio showed a near-significant exploratory association with resectional status (OR = 1.90, *p* = 0.067), whereas isolated FT3 lost statistical significance (OR = 1.69, *p* = 0.341). The model combining the FT3/FT4 ratio and CA 19-9 showed numerically the lowest AIC among the tested multivariable models (AIC = 96.0; pseudo-R^2^ = 0.324) compared with the FT3 + CA 19-9 model (AIC = 98.5; pseudo-R^2^ = 0.305) and the FT3 + FT4 + CA 19-9 model (AIC = 100.0; pseudo-R^2^ = 0.309) (Table 6B). These model comparisons should be interpreted as exploratory and should not be considered evidence of a clinically actionable prediction model.

Formal ROC analysis showed comparable exploratory discrimination of FT3 and the FT3/FT4 ratio for resectional versus non-resectional status (AUC 0.675 vs. 0.720), and the bootstrap comparison of AUC values was not statistically significant (*p* = 0.24). The FT3/FT4 ratio showed numerically lower AIC values in the univariable model (126.2 vs. 128.3) and in the model adjusted for CA 19-9 (96.0 vs. 98.5), but these differences were modest. Therefore, these findings should be interpreted as exploratory and do not demonstrate definitive superiority of the FT3/FT4 ratio over FT3 alone. Rather, the ratio may provide complementary information, particularly in relation to perioperative changes in glucose and insulin (Table 6A,B and Table 7; Figure 2 and Figure 11).

## 4. Discussion

The present study demonstrates that patients with pancreatic ductal adenocarcinoma (PDAC) undergo pronounced perioperative alterations in thyroid-related parameters accompanied by substantial nutritional and metabolic deterioration. In the whole cohort, FT3 and the FT3/FT4 ratio decreased significantly, whereas TSH increased and FT4 remained largely unchanged, a pattern more consistent with stress-related disturbance of thyroid hormone homeostasis than with primary thyroid disease [5,6,7,8,9]. These endocrine changes occurred in parallel with marked declines in albumin, total protein, glucose, insulin, HbA1c, and HDL cholesterol, together with an increase in triglycerides, indicating that the thyroid-related findings were embedded in a broader catabolic and metabolic response [1,2,3,4]. Overall, these data support the interpretation that thyroid-related indices in PDAC reflect systemic host adaptation to malignancy and surgical stress rather than an isolated endocrine phenomenon (Table 3 and Figure S2) [1,2,3,4,5,6,7,8,9,10].

A key observation is that thyroid-related indices were already linked to clinical phenotype before surgery. Patients in the resectional group had higher FT3 and FT3/FT4 ratio values, together with higher albumin and lower CA 19-9 concentrations, than patients managed without resection. In addition, baseline FT3 and FT3/FT4 ratio values correlated positively with albumin and total protein and negatively with log-transformed CA 19-9 values, suggesting that peripheral thyroid hormone conversion in PDAC is closely associated with nutritional reserve and disease burden [1,2,3,4,5,6,7]. In clinical terms, lower FT3-related indices appear to identify patients with more advanced systemic impairment rather than merely reflecting local tumor presence. This interpretation is biologically plausible in the context of PDAC cachexia, where tumor-related systemic signals, pancreatic dysfunction, and progressive malnutrition interact to shape host metabolism [2,3]. These relationships were consistently reflected in our baseline comparisons and correlation analyses (Table 1 and Table 2; Figure 2, Figure 3, Figure 4 and Figure S1) [1,2,3,4]. However, because resectional status reflects anatomical resectability, disease burden, and overall patient condition, these baseline comparisons should be interpreted as exploratory associations rather than causal effects of surgical strategy. Because CA 19-9 may be affected by cholestasis, biliary obstruction, and biliary drainage status, these associations should be interpreted as reflecting the broader disease context rather than tumor burden alone.

These findings also extend previous observations from our center. In the preliminary portal-versus-peripheral study, FT3-related parameters were linked to the local neoplastic milieu in pancreatic cancer [13]. In the subsequent pilot survival analysis, a lower preoperative FT3/FT4 ratio was associated with shorter overall survival in radically resected PDAC [14]. The present study broadens that perspective by showing that thyroid-related abnormalities in PDAC are dynamic rather than static and are strongly influenced by treatment context [13,14]. At the same time, the limited pancreatic literature outside our own center remains insufficient to support a simple causal interpretation of thyroid-related variables in PDAC [15]. The study by Garajova et al. illustrates this uncertainty, as it suggested a higher prevalence of thyroid disorders in PDAC but did not demonstrate a clear outcome effect of thyroid history or levothyroxine exposure [15].

Importantly, our data do not support a simplistic interpretation of the FT3/FT4 ratio as a tumor-specific marker that normalizes after tumor removal. If that were the dominant mechanism, one would expect a consistent postoperative improvement following resection. Instead, the opposite pattern was observed in much of the cohort: FT3 and the FT3/FT4 ratio tended to decline after treatment, and this decline was also seen in non-resectional patients, including those who underwent exploratory laparotomy only. This strongly suggests that the early postoperative hormonal profile is driven predominantly by host response, catabolic stress, and postoperative metabolic remodeling rather than by the mere presence or absence of tumor tissue [7,8,9,13,14]. Our perioperative findings support this interpretation directly (Table 3 and Table 4A; Figure 5, Figure 6, Figure 7 and Figure 8).

The perioperative hormonal pattern observed in the whole cohort is broadly compatible with non-thyroidal illness syndrome (NTIS) or low-T3 syndrome [7,8,9]. Reduced FT3 with relatively preserved FT4 and an altered or inadequate pituitary response is a well-described feature of severe systemic illness and is thought to reflect changes in deiodinase activity, thyroid hormone transport, protein binding, and hypothalamic–pituitary regulation [5,6,7,8,9]. In our cohort, this endocrine pattern occurred in parallel with marked declines in albumin and total protein, which further supports the view that postoperative thyroid changes were embedded in a wider catabolic and inflammatory state [1,2,3,4,7,8]. The nearly fourfold increase in the prevalence of low-T3 syndrome after surgery is therefore biologically plausible and consistent with the broader NTIS literature [7,8,9]. However, its prognostic implications in PDAC remain to be established prospectively [7,8,14]. In our dataset, the low-T3 phenotype was clearly visible both at the population level and in subgroup analyses (Table 5A–D; Figure 9 and Figure 10) [7,8,9].

A noteworthy and initially counterintuitive finding was the normalization of low-T3 status in 10 of 11 patients who fulfilled the low-T3 definition preoperatively. This should not be interpreted as simple endocrine recovery or as evidence against the biological relevance of low-T3 syndrome. Rather, it suggests that preoperative and postoperative low-T3 phenotypes may reflect partly different clinical contexts. In our cohort, preoperative low T3 was associated with a less favorable baseline profile, including lower albumin and total protein concentrations, higher CA 19-9 levels, and a lower FT3/FT4 ratio. By contrast, most postoperative low-T3 cases developed de novo after surgery, supporting the interpretation that early postoperative thyroid alterations were driven mainly by acute surgical stress, catabolism, inflammatory response, and procedure-specific metabolic remodeling. Given the small number of patients with preoperative low-T3 syndrome, this observation should be regarded as exploratory and requires prospective validation [7,8,9].

An especially relevant aspect of this study is that the endocrine response was not uniform across procedures. The broad comparison between resectional and non-resectional treatment showed only limited differences, whereas the four-group analysis revealed heterogeneity for thyroid-related and metabolic changes. These procedure-specific differences should be interpreted cautiously. Although they are consistent with prior observations that glucose metabolism and endocrine adaptation differ by type of pancreatic surgery, they should not be interpreted as direct mechanistic effects of the procedures themselves. The surgical groups differed clinically by definition, including tumor location, extent of pancreatic resection, splenectomy status, baseline disease burden, nutritional trajectory, and postoperative course. Therefore, the observed trajectories most likely reflect a combination of surgical anatomy, disease biology, perioperative stress, and patient selection rather than isolated effects of a given procedure (Table 4B and Figure 6, Figure 7 and Figure 8) [4,16].

The differences between the Whipple and distal pancreatectomy with splenectomy subgroups are therefore best viewed as descriptive and hypothesis-generating. In the Whipple subgroup, FT3 remained relatively stable, and the FT3/FT4 ratio did not decline significantly despite marked decreases in albumin and glucose, whereas distal pancreatectomy with splenectomy showed the most pronounced decline in FT3 and the FT3/FT4 ratio and the highest postoperative prevalence of low-T3 syndrome. These contrasts may relate to differences in patient selection, tumor location, pancreatic tissue removal, splenectomy status, postoperative inflammatory response, residual tumor burden, and baseline systemic reserve. Given the modest subgroup sizes and absence of fully adjusted procedure-specific models, these findings should not be interpreted as evidence of procedure-driven mechanisms but rather as exploratory signals requiring prospective validation (Table 4B and Figure 6, Figure 7, Figure 8 and Figure 9) [1,2,3,4,7,8,9,16].

Another important observation is that the FT3/FT4 ratio may provide complementary information to isolated FT3 measurement in selected analytical settings [5,6,10,14,18]. Although both markers were associated with resectability in univariable analysis, the FT3/FT4 ratio showed only numerically higher discriminatory performance than FT3 alone, and the difference in AUC was not statistically significant. Similarly, the AIC differences between models were modest, and the association of the FT3/FT4 ratio in the CA 19-9-adjusted model reached only trend-level significance. Therefore, these findings should not be interpreted as evidence of definitive superiority of the FT3/FT4 ratio over FT3. Rather, they suggest that the ratio may capture an additional component of thyroid-metabolic adaptation, particularly in relation to perioperative changes in glucose and insulin, and should be considered hypothesis-generating [5,6]. This interpretation remains biologically plausible because the ratio more directly reflects peripheral conversion of T4 to T3 and may therefore capture subtle changes in deiodinase activity that are not evident from the absolute FT3 concentration alone [5,6]. It is also consistent with the broader oncology literature, in which thyroid hormone ratios have shown prognostic relevance beyond isolated hormone measurements [14,18]. In particular, Pasqualetti et al. demonstrated independent prognostic significance of the FT3/FT4 ratio in advanced metastatic colorectal cancer, suggesting that this metric may capture a clinically meaningful component of host metabolic reserve in oncology [18]. In our study, these features were reflected by the regression, AUC, and comparative model analyses, but they should be interpreted as exploratory rather than confirmatory (Table 6A,B and Table 7; Figure 2 and Figure 11) [14,18].

The logistic regression and ROC analyses should also be interpreted as exploratory association analyses rather than as clinically actionable prediction models. Resectability in PDAC is primarily determined by anatomical tumor stage, vascular involvement, metastatic disease, patient condition, and multidisciplinary surgical decision making. Therefore, thyroid-related indices cannot be considered independent determinants of resectability. The observed associations of FT3 and the FT3/FT4 ratio with resectional status most likely reflect broader differences in disease burden, nutritional reserve, and systemic metabolic condition. This interpretation is further supported by the strong contribution of CA 19-9, which may itself act as a surrogate of tumor burden and disease severity.

Our findings also fit with the broader concept that thyroid-related markers in oncology are better understood as indicators of host response than as classical tumor-specific biomarkers [10,11,19,20]. Reviews of thyroid hormone signaling in cancer have emphasized that these pathways may influence proliferation, angiogenesis, invasion, apoptosis, and metabolic programming, but they also highlight that circulating thyroid-related parameters integrate the effects of systemic illness and host adaptation [10,11,19,20]. Clinical observations outside PDAC similarly suggest that lower T3 or altered FT3/FT4-related balance is associated with poorer outcomes in several malignancies, including gastroesophageal cancer and hepatocellular carcinoma [21,22]. At the same time, the exact clinical meaning of these markers appears to vary across tumor types and disease settings [10,11,18,19,20,21,22,23,24,25,26,27,28,29,30,31]. Studies in colorectal cancer, for example, have yielded mixed epidemiologic signals, ranging from apparently protective associations in some treated or population-based settings to neutral results in meta-analytic or Mendelian-randomization analyses [18,25,26,27,28,29,30]. For this reason, the present results are probably best interpreted within a host-response framework rather than as evidence that a single thyroid-derived biomarker directly tracks tumor biology in a uniform way across cancers [10,11,19,20].

The clinical implications of this study should therefore be framed carefully. At present, our findings do not justify using FT3 or the FT3/FT4 ratio for immediate perioperative risk stratification, as standalone tumor markers, or as clinically actionable predictors in PDAC [10,14,15]. Survival, postoperative morbidity, complication severity, and long-term recovery were not evaluated in the present study. However, the results suggest that these parameters may be useful as accessible markers of systemic metabolic adaptation and perioperative metabolic vulnerability, particularly when interpreted together with albumin, total protein, and CA 19-9 [1,2,3,4,14]. Such an approach should be regarded as supporting further prospective investigation of perioperative risk phenotyping and postoperative metabolic assessment rather than direct clinical implementation [1,2,3,4,7,14]. The fact that the ratio correlated not only with baseline nutritional and tumor-burden variables but also with perioperative metabolic changes further supports this clinically integrative interpretation (Table 1, Table 2, Table 3 and Table 7; Figure 3, Figure 4 and Figure S2).

This study has several strengths. First, it includes a clinically relevant PDAC cohort with both baseline and early postoperative measurements. Second, it evaluates thyroid-related, nutritional, and metabolic variables simultaneously, enabling a broader systems-level interpretation. Third, it uses two complementary grouping strategies, which made it possible to detect both broad and procedure-specific effects [4,16]. Several limitations should also be acknowledged. The retrospective single-center design introduces a risk of selection bias and residual confounding. Sample sizes in the procedure-specific subgroups were modest, limiting statistical power and requiring caution in mechanistic interpretation. The follow-up interval was relatively short, and survival, postoperative morbidity as an outcome, complication severity in outcome models, and long-term recovery were not evaluated; therefore, the present findings cannot establish clinical utility for perioperative risk stratification. We also did not measure inflammatory cytokines, reverse T3, pancreatic exocrine function directly in this cohort, or tissue-level deiodinase activity, so mechanistic conclusions remain inferential [5,6,7,8,9]. Finally, although the FT3/FT4 ratio is a practical surrogate of peripheral conversion, it does not directly capture tissue-specific thyroid hormone signaling [5,6].

An important additional limitation is the possibility of residual confounding related to differences between resectional and non-resectional patients. Although several available markers of systemic condition and disease burden, including albumin, total protein, CA 19-9, glucose, insulin, HbA1c, lipid parameters, and selected comorbidities, were analyzed, these variables do not fully capture the clinical complexity of PDAC surgical management. The groups may have differed not only in surgical strategy but also in anatomical disease stage, tumor location, biliary obstruction or cholestasis, inflammatory burden, cachexia severity, performance status, nutritional support, perioperative infections, length of hospital stay, and other factors that were not consistently available in a standardized format in the retrospective dataset. Therefore, the observed associations between thyroid-related indices, metabolic parameters, and surgical management should be interpreted as exploratory and hypothesis-generating rather than causal.

CA 19-9 concentrations should also be interpreted cautiously because they may be influenced by biliary obstruction, cholestasis, and biliary drainage status. In the present retrospective dataset, bilirubin levels and detailed information on preoperative biliary drainage were not consistently available in a standardized format and therefore could not be included in adjusted analyses. Consequently, CA 19-9 was interpreted as a marker of broader disease context and systemic burden rather than as a tumor-specific marker alone.

Procedure-specific comparisons were also particularly susceptible to confounding by tumor location, extent of pancreatic tissue removed, splenectomy status, baseline disease burden, nutritional trajectory, and postoperative course. Because of the limited sample size and retrospective design, fully adjusted procedure-specific models could not be performed reliably; therefore, the four-group findings should be considered descriptive and hypothesis-generating.

Although the final analytic cohort was restricted to patients with postoperative complication severity not exceeding Clavien–Dindo grade IIIa, detailed grade-by-grade postoperative complication data were not available for modelling. Therefore, the influence of complication severity within the included range on postoperative thyroid-metabolic changes could not be assessed. In addition, data on whether patients initiated systemic oncological treatment, including adjuvant or palliative chemotherapy, before the 4–6-week follow-up laboratory assessment were not available in the final analytic dataset, and this potential influence could not be evaluated.

Missing postoperative laboratory measurements may also have been informative rather than completely random, particularly if follow-up testing was affected by postoperative morbidity, readmission outside the study center, delayed outpatient follow-up, loss to follow-up, or other clinical factors.

Although levothyroxine therapy was considered acceptable only when stable and no documented amiodarone or systemic glucocorticoid use was identified, exposure to iodine-containing contrast could not be reliably excluded. Therefore, a potential influence of contrast-related iodine exposure on thyroid-related laboratory parameters cannot be completely ruled out.

Because reverse T3 was not measured, the low-T3 phenotype observed in this study should be interpreted as compatible with, but not diagnostic of, non-thyroidal illness syndrome. This limitation further supports treating the low-T3 analyses as exploratory and hypothesis-generating.

The regression and ROC analyses were exploratory and were not intended to provide a validated clinical prediction model for resectability. Model calibration and optimism-corrected validation were not performed because of the limited sample size and retrospective design. Therefore, these findings should be interpreted as hypothesis-generating associations with resectional status requiring external validation.

Although Benjamini–Hochberg false discovery rate adjustment was applied to the principal families of paired pre-vs-post tests, four-group Kruskal–Wallis tests, and Spearman correlations, and Dunn–Bonferroni correction was applied to post hoc pairwise comparisons, the overall study still includes numerous secondary and exploratory analyses. Therefore, statistically significant findings outside the primary endpoint should be interpreted cautiously and considered hypothesis-generating. Additional limitations include the retrospective assessment of thyroid-active medication exposure, the limited availability of detailed postoperative complication data, and the absence of validation against survival, postoperative morbidity, or long-term recovery outcomes.

Overall, the present study indicates that perioperative changes in FT3 and the FT3/FT4 ratio are common in PDAC and are closely intertwined with nutritional and metabolic deterioration [1,2,3,4,7,8,9]. Their baseline values and early postoperative dynamics appear to reflect disease burden and systemic host response more convincingly than tumor-specific activity alone [1,2,3,4,10,13,14,15]. This interpretation provides a coherent biological framework for future studies of thyroid hormone homeostasis in pancreatic cancer [10,11,13,14,15,19,20]. Prospective studies with larger cohorts, longer follow-up, and integrated inflammatory, nutritional, endocrine, and clinical outcome assessment are now needed before these indices can be considered for perioperative risk stratification or postoperative outcome assessment in PDAC [1,2,3,4,7,8,14].

## 5. Conclusions

In conclusion, patients with PDAC exhibited significant perioperative alterations in thyroid hormone homeostasis, particularly in FT3 and the FT3/FT4 ratio, which occurred in parallel with marked nutritional and metabolic deterioration. These changes were not uniform across surgical strategies and became much more apparent when patients were analyzed according to specific procedure type rather than only as resectional versus non-resectional cases. Baseline FT3-related indices were associated with albumin, total protein, and CA 19-9, supporting their relationship with nutritional reserve and disease burden. Moreover, the FT3/FT4 ratio may provide complementary insight to FT3 alone, particularly because it showed associations with perioperative metabolic adaptation and numerically favorable but not statistically definitive performance in exploratory resectional-status analyses. Taken together, these findings suggest that FT3 and the FT3/FT4 ratio should be interpreted in PDAC primarily as markers of systemic host response and thyroid-metabolic adaptation rather than as tumor-specific biomarkers or clinically actionable predictors of resectability. Because survival, postoperative morbidity, complication severity, and long-term recovery were not evaluated, the present findings should be viewed as supporting further investigation rather than constituting immediate clinical implementation. Further prospective studies incorporating established clinical, nutritional, inflammatory, oncological, and outcome variables are needed to determine whether these indices may contribute to perioperative phenotyping or risk assessment in pancreatic cancer.

## Figures and Tables

**Table 1:** Baseline characteristics of patients (resection vs. non-resection). Values are median [Q1–Q3]. Tests: Mann–Whitney U for continuous variables and Fisher’s exact test for categorical variables.

Variable	Resection (*n* = 49)	Non-Resection (*n* = 52)	*p*
Age [years]	64.0 [59.0–71.0]	68.5 [56.5–77.0]	0.384
FT3 [pg/mL]	2.8 [2.5–3.0]	2.4 [2.2–2.8]	0.003
FT4 [ng/dL]	1.1 [1.1–1.5]	1.4 [1.2–1.6]	0.002
TSH [mIU/L]	1.0 [0.6–1.4]	0.8 [0.6–1.3]	0.545
FT3/FT4 ratio	2.4 [1.8–2.8]	1.7 [1.2–2.2]	<0.001
Albumin [g/L]	42.7 [41.6–44.6]	38.0 [35.7–42.0]	0.001
Total protein [g/L]	71.0 [62.6–73.0]	62.4 [58.5–69.4]	0.031
Glucose [mg/dL]	112.0 [98.0–133.0]	122.5 [97.0–143.2]	0.516
Insulin [μIU/mL]	7.3 [5.3–11.5]	8.9 [5.0–12.1]	0.697
HbA1c [%]	5.5 [5.4–6.0]	5.8 [5.5–6.6]	0.024
Cholesterol [mg/dL]	180.0 [130.8–215.8]	170.0 [127.2–195.5]	0.462
LDL [mg/dL]	88.5 [58.8–131.8]	99.0 [65.5–125.2]	0.812
HDL [mg/dL]	45.0 [37.0–56.5]	37.5 [30.2–49.0]	0.028
TG [mg/dL]	84.0 [61.0–136.0]	114.5 [82.5–166.0]	0.011
CA 19-9 [U/mL]	7.9 [1.6–22.1]	77.7 [29.5–239.2]	<0.001
Diabetes, *n* (%)	11 (22%)	19 (37%)	0.134
Thyroid disease, *n* (%)	10 (20%)	10 (19%)	1.000
Hypertension, *n* (%)	28 (57%)	30 (58%)	1.000
Dyslipidemia, *n* (%)	3 (6%)	9 (17%)	0.124

**Table 2:** Spearman correlations (ρ) between thyroid-related parameters and baseline characteristics.

Outcome Variable	FT3 (ρ)	*p*	FT3/FT4 Ratio ρ [95% CI]	*p*
Albumin	+0.356	<0.001	+0.212 [+0.011, +0.390]	0.037
Total protein	+0.283	0.005	+0.206 [+0.002, +0.402]	0.044
log(CA 19-9)	−0.320	0.001	−0.329 [−0.512, −0.135]	<0.001
Age	−0.285	0.004	−0.238 [−0.439, −0.017]	0.018
Glucose	−0.156	0.123	—	—
Insulin	−0.043	0.674	—	—
HbA1c	+0.011	0.914	—	—
HDL	+0.192	0.074	—	—
TG	−0.124	0.243	—	—

**Table 3:** Perioperative changes in the whole cohort (paired Wilcoxon signed-rank test). Values are median [IQR]. Δ = median change (postoperative − preoperative).

Variable	*n*	Before	After	Δ Median [95% CI]	*p*
FT3 [pg/mL]	93	2.6 [0.8]	2.0 [1.0]	−0.65 [−0.74, −0.48]	<0.001
FT4 [ng/dL]	93	1.3 [0.5]	1.2 [0.4]	−0.04 [−0.14, +0.05]	0.110
TSH [mIU/L]	94	0.9 [0.7]	1.4 [1.7]	+0.28 [+0.10, +0.64]	<0.001
FT3/FT4 ratio	93	2.1 [1.1]	1.5 [0.8]	−0.43 [−0.55, −0.18]	<0.001
Albumin [g/L]	92	41.6 [6.5]	32.0 [9.3]	−8.65 [−10.00, −6.80]	<0.001
Total protein [g/L]	90	66.9 [12.5]	58.5 [15.3]	−7.10 [−10.00, −5.05]	<0.001
Glucose [mg/dL]	100	118.5 [44.0]	99.5 [40.2]	−14.50 [−21.50, −6.50]	<0.001
Insulin [μIU/mL]	96	7.9 [7.1]	5.2 [4.4]	−1.26 [−3.00, −0.45]	<0.001
HbA1c [%]	95	5.7 [0.8]	5.7 [1.1]	−0.20 [−0.30, −0.10]	<0.001
HDL [mg/dL]	84	40.5 [19.5]	32.5 [15.0]	−8.00 [−9.00, −4.00]	<0.001
TG [mg/dL]	85	104.0 [69.0]	145.0 [113.0]	+36.00 [+11.00, +62.00]	<0.001
Cholesterol [mg/dL]	85	170.0 [74.0]	150.0 [78.0]	−12.00 [−25.00, −8.00]	0.011
LDL [mg/dL]	84	96.0 [63.0]	80.0 [59.8]	−7.50 [−18.00, +4.50]	0.080

**Table 4:** (**A**) Comparison of perioperative changes between resectional and non-resectional treatment. Values represent median perioperative change, defined as postoperative minus preoperative value. Between-group comparisons were performed using the Mann–Whitney U test. (**B**) Procedure-specific comparison of perioperative changes across four surgical groups. Values represent median perioperative change, defined as postoperative minus preoperative value. Comparisons across groups were performed using the Kruskal–Wallis test. Groups included Whipple procedure, distal pancreatectomy with splenectomy, palliative bypass, and exploratory laparotomy.

(**A**)					
**ΔVariable**	**Resection**		**Non-Resection**		***p***
ΔFT3	−0.67		−0.65		0.110
ΔFT4	+0.05		−0.10		0.328
ΔTSH	+0.08		+0.47		0.114
ΔFT3/FT4	−0.52		−0.34		0.188
ΔAlbumin	−9.50		−8.00		0.749
ΔProtein	−7.10		−7.80		0.977
ΔGlucose	−14.50		−13.50		0.748
ΔInsulin	−1.90		−1.10		0.404
ΔHbA1c	−0.10		−0.20		0.161
ΔHDL	−9.00		−6.50		0.498
ΔTG	+72.00		+4.50		0.003
(**B**)					
**ΔVariable**	**Whipple** **(*n* = 22)**	**Distal Pancreatectomy with Splenectomy** **(*n* = 27)**	**Palliative Bypass** **(*n* = 25)**	**Exploratory** **Laparotomy** **(*n* = 27)**	***p *** **(K–W)**
ΔFT3	−0.17	−1.15	−0.69	−0.39	<0.001
ΔFT4	−0.10	+0.07	−0.23	+0.05	0.003
ΔTSH	+1.27	−0.31	+0.52	+0.32	<0.001
ΔFT3/FT4	+0.03	−0.86	−0.24	−0.51	<0.001
ΔAlbumin	−13.35	−9.00	−9.50	−7.00	0.086
ΔGlucose	−38.00	−13.00	−34.00	+1.00	<0.001
ΔInsulin	−1.35	−2.90	−5.10	+0.08	<0.001
ΔHDL	−12.00	−8.00	−5.00	−8.00	0.498
ΔTG	+79.00	+70.00	+5.00	+4.00	0.027

**Table 5:** (**A**) Prevalence of low-T3 syndrome (FT3 < 2.0 pg/mL, TSH 0.3–4.0 mIU/L) before and after surgery. (**B**) Transition table for low-T3 status (before → after surgery). (**C**). Distribution of postoperative low-T3 syndrome by surgical group. (**D**). Baseline profile of patients with preoperative low-T3 syndrome vs. euthyroid patients.

(**A**)					
**Time Point**	**Low T3**	***n***			**%**
Before surgery	11	99			11.1%
After surgery (follow-up)	36	93			38.7%
(**B**)					
	**Low T3 After**		**Normal After**		
Low-T3 before	1		10		
Normal before	35		47		
(**C**)					
**Group**	**Low T3**	***n***		**%**	
Whipple	1	20		5.0%	
Distal pancreatectomy with splenectomy	13	24		54.2%	
Palliative bypass	13	25		52.0%	
Exploratory laparotomy	9	24		37.5%	
(**D**)					
**Parameter**	**Low T3 (*n* = 11)**	**Normal (*n* = 86)**		***p***	
Albumin [g/L]	36.0	41.6		0.003	
Total protein [g/L]	62.0	69.0		0.006	
CA 19-9 [U/mL]	95.7	21.0		0.020	
FT3/FT4 ratio	1.2	2.2		<0.001	

McNemar’s test: χ^2^ = 12.80; *p* < 0.001.

**Table 6:** (**A**) Univariable logistic regression for exploratory associations with resectional status. (**B**) Multivariable logistic regression models for exploratory associations with resectional status. OR—odds ratio; CI—confidence interval; pseudo-R^2^—pseudo coefficient of determination; NS—not significant.

(**A**)						
**Predictor**	**OR**		**95% CI**	***p***	**AIC**	
FT3 [pg/mL]	3.65		1.45–9.18	0.006	128.3	
FT3/FT4 ratio	2.57		1.39–4.74	0.003	126.2	
Albumin [g/L]	1.09		1.00–1.18	0.038	132.3	
log(CA 19-9)	0.40		0.27–0.58	<0.001	97.4	
Age	0.99		0.96–1.03	0.786	137.0	
(**B**)						
**Model**	**Predictor**	**OR**	**95% CI**	***p***	**AIC**	**Pseudo-R^2^**
FT3/FT4 ratio + CA 19-9	FT3/FT4 ratio	1.90	0.96–3.76	0.067	96.0	0.324
	log(CA 19-9)	0.42	0.29–0.61	<0.001		
FT3 + CA 19-9	FT3	1.69	0.57–5.01	0.341	98.5	0.305
	log(CA 19-9)	0.42	0.28–0.61	<0.001		
FT3 + FT4 + CA 19-9	FT3	NS	0.49–4.68	NS	100.0	0.309
	FT4	NS	0.29–1.73	NS		
	log(CA 19-9)	0.41	0.28–0.61	<0.001		

**Table 7:** AUC and ΔAUC 95% CIs estimated by paired percentile bootstrap (5000 resamples). AUC computed on *n* = 99 (paired FT3 and FT4 available); AIC values from logistic regression models in Table 6 (*n* = 96). NS—not significant.

Criterion	FT3	FT3/FT4 Ratio
AUC (ROC)	0.675 [0.566–0.781]	0.720 [0.614–0.821]
Bootstrap ΔAUC	—	+0.045 [−0.028 to +0.120]; *p* = 0.24
Correlation between markers (ρ)	0.798	—
AIC (univariable model)	128.3	126.2
AIC (model adjusted for CA 19-9)	98.5	96.0
*p* in model adjusted for CA 19-9	0.341 (NS)	0.067 (trend)
Correlation with Δglucose	NS	*p* = 0.030
Correlation with Δinsulin	NS	*p* = 0.040

## Data Availability

The data presented in this study are available from the corresponding author upon reasonable request. The data are not publicly available due to privacy and institutional restrictions.

## Supplementary Materials

The following supporting information can be downloaded at https://www.mdpi.com/article/10.3390/cancers18111769/s1, Table S1: (A) Standardized mean differences for propensity score covariates before and after IPTW. (B) IPTW-weighted between-group differences for principal outcomes. Table S2: Raw and Benjamini–Hochberg-adjusted *p* values for paired pre-vs-post tests, four-group Kruskal–Wallis tests, and principal Spearman correlations. Table S3: Post hoc pairwise comparisons (Dunn’s test with Bonferroni correction) after significant Kruskal–Wallis tests. Table S4: Missing data per laboratory variable and time point. Figure S1: Correlation matrix of baseline thyroid-related, nutritional, metabolic, and tumor-burden parameters. Figure S2: Correlations between perioperative changes in the FT3/FT4 ratio and changes in glucose and insulin concentrations.

## Abbreviations

PDAC	pancreatic ductal adenocarcinoma
TSH	thyroid-stimulating hormone
FT3	free triiodothyronine
FT4	free thyroxine
NTIS	non-thyroidal illness syndrome
HDL	high-density lipoprotein
LDL	low-density lipoprotein
TG	triglyceride
HbA1c	glycated hemoglobin
AIC	Akaike information criterion
ROC	receiver operating characteristic
AUC	area under the curve
