# Supplementary material for: Perioperative Thyroid-Metabolic Changes in Pancreatic Ductal Adenocarcinoma According to Surgical Management
Source: Cancers (Basel). 2026 May 28;18(11):1769. doi: 10.3390/cancers18111769 (PMC13255950; doi:10.3390/cancers18111769)
Supplement: Supplementary file 1 [file cancers-18-01769-s001.zip › cancers-4311091-supplementary.pdf]

Supplementary Materials

Perioperative thyroid-metabolic changes in patients with pancreatic ductal adenocarcinoma undergoing resection, palliative bypass, or exploratory laparotomy

Table S1. (**A**) Standardized mean differences (SMD) for propensity score covariates before and after IPTW. (**B**) Crude and IPTW-weighted between-group differences (Resection − Non-resection) for principal outcomes.

**Covariate**	**SMD (raw)**	**SMD (IPTW)**
Age	-0.12	+0.02
Diabetes	-0.31	+0.01
Hypertension	-0.01	+0.01
Thyroid disease	+0.03	-0.04
Dyslipidemia	-0.35	+0.01
**Outcome**	**Crude difference**	**IPTW-weighted difference**
FT3 baseline (pg/mL)	+0.30	+0.33
FT3/FT4 ratio baseline	+0.54	+0.58
Albumin baseline (g/L)	+2.56	+2.70
log(CA 19-9) baseline	-2.36	-2.43
Delta FT3	-0.24	-0.21
Delta FT3/FT4 ratio	-0.30	-0.29
Delta TG (mg/dL)	+49.21	+48.60

$|SMD| < 0.10$ indicates adequate covariate balance. Inverse-probability-of-treatment weights truncated at the 1st and 99th percentile. Crude differences from the unweighted analytic dataset; IPTW-weighted differences from the inverse-probability-of-treatment weighted pseudopopulation. The direction and magnitude of all contrasts are essentially unchanged after weighting.

Table S2. Raw and Benjamini–Hochberg false discovery rate (BH-FDR)-adjusted p values across three families of related tests.

Panel A. Paired pre-vs-post Wilcoxon signed-rank tests (whole cohort).

Variable	n	Δ median	p (raw)	p (BH-FDR)
Albumin	92	-8.65	< 1e-10	< 1e-10
FT3	93	-0.65	< 1e-10	3.87e-10
HDL	84	-8.00	1.26e-07	5.03e-07
FT3/FT4 ratio	93	-0.43	6.97e-07	2.09e-06
HbA1c	95	-0.20	1.33e-06	3.20e-06
Insulin	96	-1.26	1.87e-05	3.74e-05
TSH	94	+0.28	3.04e-05	4.79e-05
TG	85	+36.00	3.19e-05	4.79e-05
Glucose	100	-14.50	0.000170	0.000226
Cholesterol	85	-12.00	0.010552	0.012663
LDL	84	-7.50	0.079555	0.086787
FT4	93	-0.04	0.109639	0.109639

Panel B. Kruskal–Wallis tests across four surgical groups.

Variable	n	p (raw)	p (BH-FDR)
ΔInsulin	96	4.94e-06	5.44e-05
ΔTSH	94	7.39e-05	0.000406
ΔGlucose	100	0.000209	0.000663
ΔFT3	93	0.000241	0.000663
ΔFT3/FT4 ratio	93	0.000475	0.001044
ΔFT4	93	0.003192	0.005852
ΔTG	85	0.027364	0.043001

ΔAlbumin	92	0.086448	0.118866
ΔHbA1c	95	0.214166	0.261759
ΔTotal protein	90	0.335390	0.368929
ΔHDL	84	0.524752	0.524752

Panel C. Principal Spearman rank correlations.

Correlation	n	Spearman ρ	p (raw)	p (BH-FDR)
FT3 vs Albumin	97	+0.356	0.000347	0.003820
FT3/FT4 vs log(CA 19-9)	98	-0.329	0.000943	0.004793
FT3 vs log(CA 19-9)	98	-0.320	0.001307	0.004793
FT3 vs Age	99	-0.285	0.004314	0.011329
FT3 vs Total protein	96	+0.283	0.005150	0.011329
FT3/FT4 vs Age	99	-0.238	0.017811	0.032654
Δ FT3/FT4 vs Δ Glucose	93	-0.225	0.029952	0.047068
FT3/FT4 vs Albumin	97	+0.212	0.037304	0.048473
ΔFT3/FT4 vs Δ Insulin	88	-0.219	0.040075	0.048473
FT3/FT4 vs Total protein	96	+0.206	0.044066	0.048473
ΔFT3/FT4 vs Δ Albumin	90	-0.172	0.104864	0.104864

BH-FDR correction applied within each family of related tests. Within each panel, p_BH < 0.05 corresponds to control of the family-wise false discovery rate at 5%.

Table S3. Post-hoc pairwise comparisons (Dunn's test with Bonferroni correction) after significant Kruskal–Wallis tests.

Variable	Group 1	Group 2	n_1	n_2	z	p (raw)	p (Bonferroni)
ΔInsulin	Whipple	Distal+splenectomy	17	27	-0.78	0.4336	1.0000
ΔInsulin	Whipple	Palliative bypass	17	25	-3.36	0.0008	0.0047
ΔInsulin	Whipple	Exploratory	17	27	+1.16	0.2443	1.0000
ΔInsulin	Distal+splenectomy	Palliative bypass	27	25	-2.93	0.0034	0.0203
ΔInsulin	Distal+splenectomy	Exploratory	27	27	+2.21	0.0267	0.1604
ΔInsulin	Palliative bypass	Exploratory	25	27	+5.10	3.33e-07	2.00e-06
ΔTSH	Whipple	Distal+splenectomy	20	25	-4.35	1.37e-05	8.21e-05
ΔTSH	Whipple	Palliative bypass	20	25	-1.06	0.2885	1.0000
ΔTSH	Whipple	Exploratory	20	24	-1.59	0.1120	0.6719
ΔTSH	Distal+splenectomy	Palliative bypass	25	25	+3.49	0.0005	0.0029
ΔTSH	Distal+splenectomy	Exploratory	25	24	+2.88	0.0040	0.0237
ΔTSH	Palliative bypass	Exploratory	25	24	-0.57	0.5689	1.0000
ΔGlucose	Whipple	Distal+splenectomy	22	26	+2.44	0.0146	0.0876
ΔGlucose	Whipple	Palliative bypass	22	25	-0.72	0.4702	1.0000
ΔGlucose	Whipple	Exploratory	22	27	+2.81	0.0049	0.0293
ΔGlucose	Distal+splenectomy	Palliative bypass	26	25	-3.28	0.0010	0.0062
ΔGlucose	Distal+splenectomy	Exploratory	26	27	+0.37	0.7131	1.0000
ΔGlucose	Palliative bypass	Exploratory	25	27	+3.67	0.0002	0.0014
ΔFT3	Whipple	Distal+splenectomy	20	24	-3.93	8.37e-05	0.0005
ΔFT3	Whipple	Palliative bypass	20	25	-1.57	0.1155	0.6931
ΔFT3	Whipple	Exploratory	20	24	-0.51	0.6075	1.0000
ΔFT3	Distal+splenectomy	Palliative bypass	24	25	+2.52	0.0119	0.0714
ΔFT3	Distal+splenectomy	Exploratory	24	24	+3.59	0.0003	0.0020

ΔFT3	Palliative bypass	Exploratory	25	24	+1.11	0.2678	1.0000
ΔFT3/FT4 ratio	Whipple	Distal+splenectomy	20	24	-3.53	0.0004	0.0025
ΔFT3/FT4 ratio	Whipple	Palliative bypass	20	25	-0.14	0.8851	1.0000
ΔFT3/FT4 ratio	Whipple	Exploratory	20	24	-1.94	0.0527	0.3163
ΔFT3/FT4 ratio	Distal+splenectomy	Palliative bypass	24	25	+3.59	0.0003	0.0020
ΔFT3/FT4 ratio	Distal+splenectomy	Exploratory	24	24	+1.67	0.0941	0.5647
ΔFT3/FT4 ratio	Palliative bypass	Exploratory	25	24	-1.90	0.0573	0.3440
ΔFT4	Whipple	Distal+splenectomy	20	24	+0.63	0.5264	1.0000
ΔFT4	Whipple	Palliative bypass	20	25	-1.98	0.0482	0.2890
ΔFT4	Whipple	Exploratory	20	24	+1.37	0.1705	1.0000
ΔFT4	Distal+splenectomy	Palliative bypass	24	25	-2.75	0.0060	0.0363
ΔFT4	Distal+splenectomy	Exploratory	24	24	+0.77	0.4395	1.0000
ΔFT4	Palliative bypass	Exploratory	25	24	+3.53	0.0004	0.0025
ΔTG	Whipple	Distal+splenectomy	19	22	+0.13	0.8941	1.0000
ΔTG	Whipple	Palliative bypass	19	22	-2.15	0.0316	0.1897
ΔTG	Whipple	Exploratory	19	22	-1.88	0.0603	0.3618
ΔTG	Distal+splenectomy	Palliative bypass	22	22	-2.37	0.0178	0.1065
ΔTG	Distal+splenectomy	Exploratory	22	22	-2.09	0.0366	0.2199
ΔTG	Palliative bypass	Exploratory	22	22	+0.28	0.7787	1.0000

Dunn's test (z statistic, two-sided) with Bonferroni correction across the six pairwise contrasts within each family. Bonferroni-adjusted $p < 0.05$ indicates a significant pairwise difference.

Table S4. Missing data per laboratory variable and time point (N = 101).

Variable	Timepoint	Available, n	Missing, n	Missing, %
TSH [mIU/L]	Baseline	99	2	2.0%
TSH [mIU/L]	Follow-up (4–6 wk)	94	7	6.9%
FT3 [pg/mL]	Baseline	99	2	2.0%
FT3 [pg/mL]	Follow-up (4–6 wk)	93	8	7.9%
FT4 [ng/dL]	Baseline	99	2	2.0%
FT4 [ng/dL]	Follow-up (4–6 wk)	93	8	7.9%
Albumin [g/L]	Baseline	97	4	4.0%
Albumin [g/L]	Follow-up (4–6 wk)	92	9	8.9%
Total protein [g/L]	Baseline	96	5	5.0%
Total protein [g/L]	Follow-up (4–6 wk)	90	11	10.9%
Glucose [mg/dL]	Baseline	101	0	0.0%
Glucose [mg/dL]	Follow-up (4–6 wk)	100	1	1.0%
Insulin [µIU/mL]	Baseline	99	2	2.0%
Insulin [µIU/mL]	Follow-up (4–6 wk)	96	5	5.0%
HbA1c [%]	Baseline	97	4	4.0%
HbA1c [%]	Follow-up (4–6 wk)	95	6	5.9%
Total cholesterol [mg/dL]	Baseline	90	11	10.9%
Total cholesterol [mg/dL]	Follow-up (4–6 wk)	85	16	15.8%
LDL [mg/dL]	Baseline	88	13	12.9%
LDL [mg/dL]	Follow-up (4–6 wk)	84	17	16.8%
HDL [mg/dL]	Baseline	88	13	12.9%
HDL [mg/dL]	Follow-up (4–6 wk)	84	17	16.8%
Triglycerides [mg/dL]	Baseline	91	10	9.9%

Triglycerides [mg/dL]	Follow-up (4–6 wk)	85	16	15.8%
CA 19-9 [U/mL]	Baseline	99	2	2.0%
CA 19-9 [U/mL]	Follow-up (4–6 wk)	68	33	32.7%

Baseline = within 7 days preoperatively; Follow-up = 4–6 weeks postoperatively. Available-case analysis was used for each test.

Figure S1. Correlation matrix of baseline thyroid-related, nutritional, metabolic, and tumor-burden parameters. Colors and circle size indicate the direction and magnitude of Spearman's rank correlation coefficient (ϱ). Blue indicates positive correlations, and red indicates negative correlations. Asterisks indicate statistical significance: * $p < 0.05$; ** $p < 0.01$; *** $p < 0.001$.

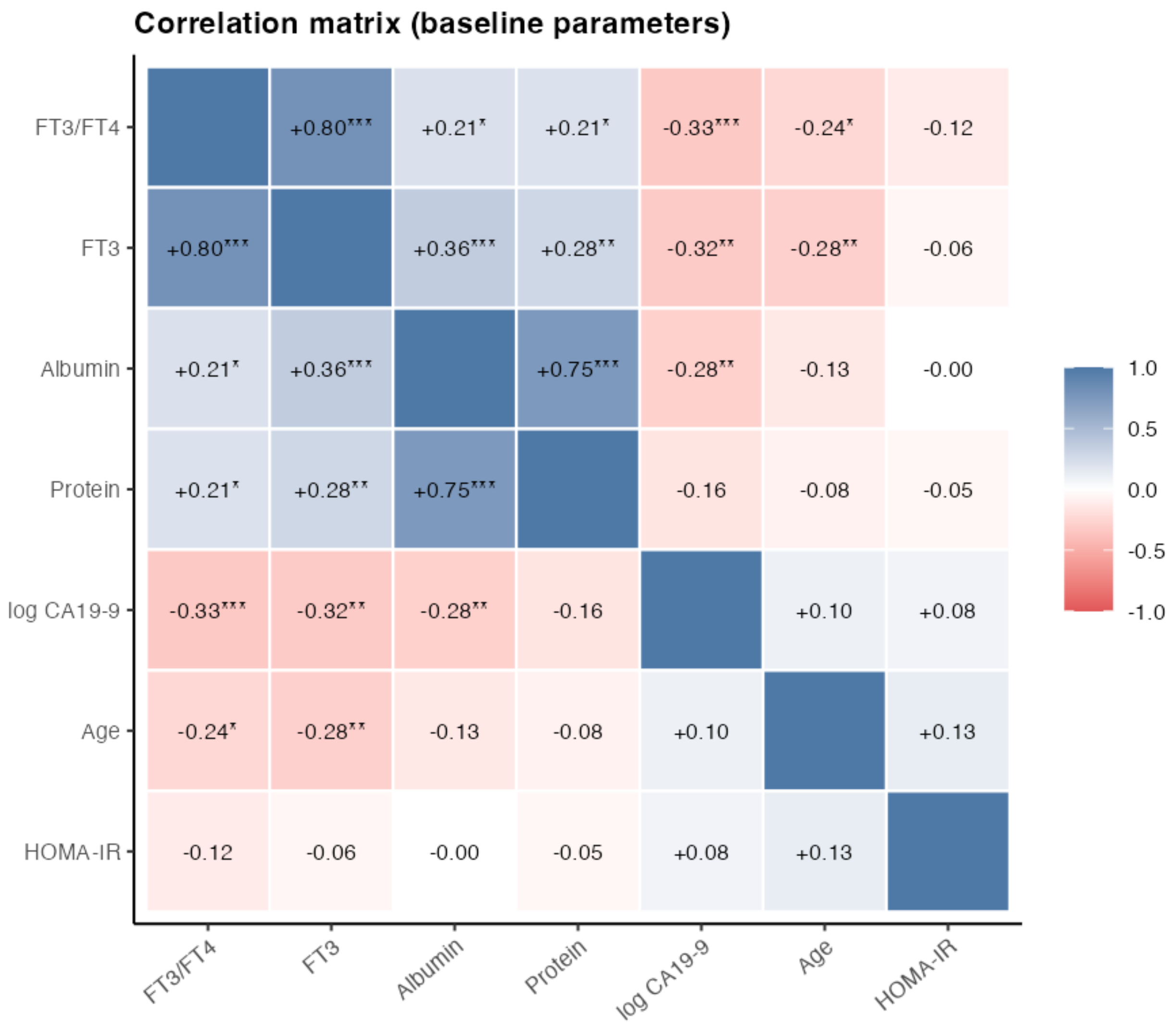

Spearman rank correlations between baseline thyroid-related parameters (TSH, FT3, FT4, FT3/FT4 ratio), nutritional parameters (albumin, total protein), metabolic parameters (glucose, insulin, HbA1c, lipid panel), and CA 19-9. Color intensity reflects the magnitude of the correlation.

Figure S2. Correlations between perioperative changes in the FT3/FT4 ratio and changes in glucose and insulin concentrations.

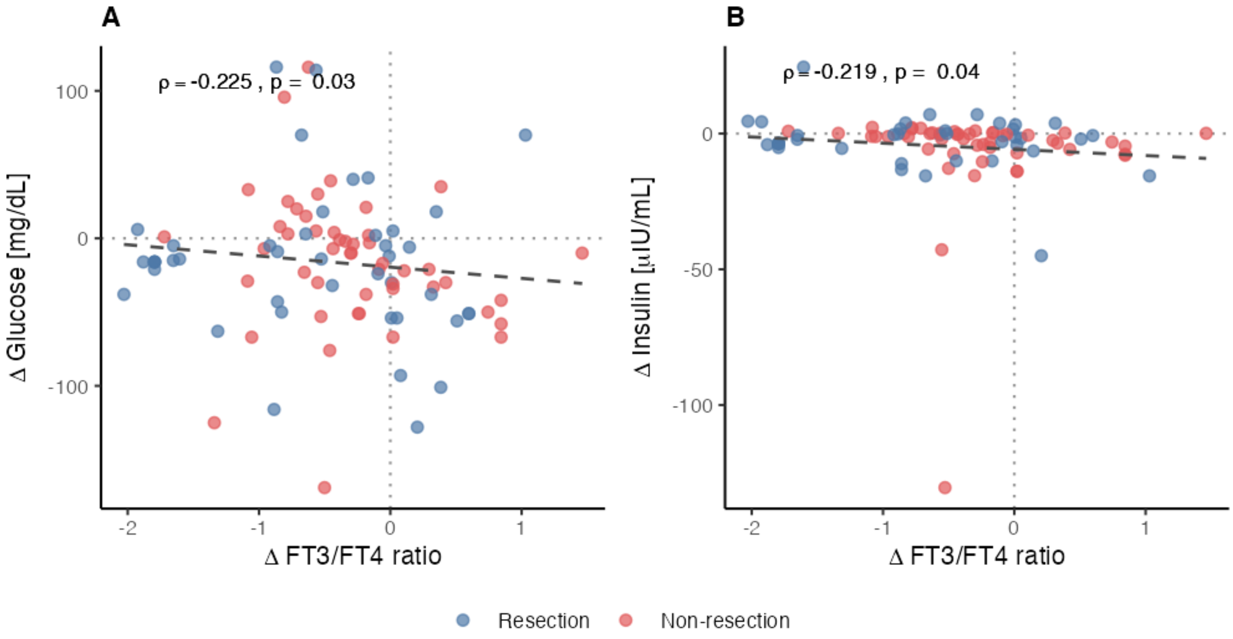

Spearman correlations between perioperative change (Δ = postoperative − preoperative) in the FT3/FT4 ratio and changes in glucose and insulin. Each dot represents one patient.
